# The Week in the Courts

**Published:** 1911-03-25

**Authors:** 


### The Week in the Courts.

RESPONSIBILITY OF OPTICIANS.

In the King's Bench Division of the High Court of Justice, before the Lord Chief Justice and a special jury, a case was heard of considerable importance to opticians. The plaintiff, a young woman who was formerly preparing to be a science teacher, suffered trouble with her eyes. She called at the shop, in Manchester, of the defendant, who carried on business as an optician and "eyesight specialist" under the name of Wood Abrahams. An assistant tested her eyes and supplied spectacles. These were not altogether satisfactory, and plaintiff returned to the shop on subsequent occasions to have her sight tested. As her condition did not improve she consulted an oculist, who found that she was suffering from conical cornea, and had evidently had the disease for several years. Plaintiff claimed damages, and it was submitted on her behalf that if the defendant had exercised reasonable care, and was possessed of reasonable skill, he must have seen that it was a case of disease and not one of ordinary short sight. Mr. Charles Wray, F.R.C.S., gave evidence on behalf of the plaintiff, and said the existence of the disease conical cornea was quite easy to discover. Mr. Harman, F.R.C.S., also gave evidence, in the course of which he said that as a rule the examination by retinoscope was quite sufficient to diagnose the disease. For the defence it was argued that defendant did not profess to be able to diagnose and cure diseases of the eye, but was simply a maker and seller of spectacles. Mr. Stephenson, F.R.C.S., Mr. Hartridge, F.R.C.S., and Dr. Ettles, called by the defendant's counsel, said that in the early stages conical cornea was difficult to detect even for a scientifically equipped man. In summing up, the Lord Chief Justice said the defendant was carrying on a well-known trade, that of an optician who sells spectacles and purports to test people's eyes for these spectacles. Therefore, if the jury thought that in the performance of his duty as an optician towards this young woman he had been guilty of negligence, undoubtedly she was entitled to damages. If they came to the conclusion that the defendant as a qualified optician from his own point of view ought to have discovered the conical cornea, then telling her that she had no disease would of course be negligence. If they came to the conclusion that he might without negligence have not found out the conical cornea, then it was only of importance if they believed that he did make that statement without proper examination so far as he was concerned. The jury found for the plaintiff, with £25 damages, and judgment, with costs, was entered accordingly.

